# A Large Proportion of *P. falciparum* Isolates in the Amazon Region of Peru Lack *pfhrp2* and *pfhrp3*: Implications for Malaria Rapid Diagnostic Tests

**DOI:** 10.1371/journal.pone.0008091

**Published:** 2010-01-25

**Authors:** Dionicia Gamboa, Mei-Fong Ho, Jorge Bendezu, Katherine Torres, Peter L. Chiodini, John W. Barnwell, Sandra Incardona, Mark Perkins, David Bell, James McCarthy, Qin Cheng

**Affiliations:** 1 Instituto de Medicina Tropical “Alexander von Humboldt” Universidad Peruana Cayetano Heredia, Lima, Peru; 2 Departamento de Bioquimica, Biologia Molecular y Farmacologia, Facultad de Ciencias, Universidad Peruana Cayetano Heredia, Lima, Peru; 3 Clinical Tropical Medicine, Queensland Institute of Medical Research, Brisbane, Queensland, Australia; 4 Hospital for Tropical Diseases, London, United Kingdom; 5 Centers for Disease Control and Prevention, Atlanta, Georgia, United States of America; 6 Foundation for Innovative New Diagnostics (FIND), Geneva, Switzerland; 7 World Health Organization - Regional Office for the Western Pacific, Manila, Philippines; 8 School of Medicine, University of Queensland, Brisbane, Queensland, Australia; 9 Drug Resistance and Diagnostics, Australian Army Malaria Institute, Brisbane, Queensland, Australia; 10 Malaria Drug Resistance and Chemotherapy, Queensland Institute of Medical Research, Brisbane, Queensland, Australia; Karolinska Institutet/Karolinska University Hospital, Sweden

## Abstract

**Background:**

Malaria rapid diagnostic tests (RDTs) offer significant potential to improve the diagnosis of malaria, and are playing an increasing role in malaria case management, control and elimination. Peru, along with other South American countries, is moving to introduce malaria RDTs as components of malaria control programmes supported by the Global Fund for AIDS, TB and malaria. The selection of the most suitable malaria RDTs is critical to the success of the programmes.

**Methods:**

Eight of nine microscopy positive *P. falciparum* samples collected in Iquitos, Peru tested negative or weak positive using HRP2-detecting RDTs. These samples were tested for the presence of *pfhrp2* and *pfhrp3* and their flanking genes by PCR, as well as the presence of HRP proteins by ELISA. To investigate for geographic extent of HRP-deleted parasites and their temporal occurrence a retrospective study was undertaken on 148 microscopy positive *P. falciparum* samples collected in different areas of the Amazon region of Peru.

**Findings:**

Eight of the nine isolates lacked the *pfhrp2* and/or *pfhrp3* genes and one or both flanking genes, and the absence of HRP was confirmed by ELISA. The retrospective study showed that 61 (41%) and 103 (70%) of the 148 samples lacked the *pfhrp2* or *pfhrp3* genes respectively, with 32 (21.6%) samples lacking both *hrp genes*.

**Conclusions:**

This is the first documentation of *P. falciparum* field isolates lacking *pfhrp2* and/or *pfhrp3*. The high frequency and wide distribution of different parasites lacking *pfhrp2* and/or *pfhrp3* in widely dispersed areas in the Peruvian Amazon implies that malaria RDTs targeting HRP2 will fail to detect a high proportion of *P. falciparum* in malaria-endemic areas of Peru and should not be used. RDTs detecting parasite LDH or aldolase and quality microscopy should be use for malaria diagnosis in this region. There is an urgent need for investigation of the abundance and geographic distribution of these parasites in Peru and neighbouring countries.

## Introduction

Despite several decades of control efforts malaria remains a major infectious disease, causing at least 250 million infections and nearly 1 million deaths per year [1]. The recent significant reductions in prevalence documented in some settings [1] through effective use of Artemisinin-based Combination Therapy (ACT), insecticide-treated bed nets and indoor residual spraying has given cause for optimism and placed malaria eradication back on the global health agenda. The ability to accurately and rapidly diagnose malaria infection in different settings is essential to the success of malaria control and elimination [2]. Accurate diagnosis facilitates appropriate and prompt treatment of febrile illness, reduces drug misuse, and minimises the risk of the development of drug resistance.

Microscopic examination of blood smears has been the traditional method for detecting malaria parasites. The accuracy of microscopy largely relies on the experience and training of the microscopists and the quality of smears. Unfortunately, the quality of microscopy varies significantly, and is often unreliable. Importantly, good quality microscopy is particularly hard to maintain in remote areas where malaria commonly occurs [3]. Rapid Diagnostic Tests (RDTs) for malaria have become increasingly recognized as essential components of efforts to improve diagnostic accuracy in areas where a large part of the population at risk of malaria.

Malaria RDTs are lateral flow devices that detect parasite proteins using antibodies. The tests are easy to perform and provide rapid results (in 15 to 20 min) without the need of electricity, expensive equipment or extensive training. RDTs have, therefore, great potential for rapid and accurate malaria diagnosis in most malaria-endemic areas. Today, over 50 brands of malaria RDTs are manufactured, with over 150 individual products being commercially available. Some detect *P. falciparum* only, while others detect *P. falciparum* and other *Plasmodium* species. Most *P. falciparum-*detecting RDTs target histidine rich protein 2 (HRP2). HRP2 is an abundant protein produced by all blood stages of *P. falciparum* [4], and is notable for a number of alanine and histidine rich repeats [5].

Peru is endemic for both *P. falciparum* and *P. vivax*, with most cases reported in the Amazon Region [6]. The rate of infection/person/season detected by active and passive case detections in Iquitos, the main city in the Peruvian Amazon region, was reported as 0.13 for *P. falciparum* and 0.39 for *P. vivax* in 2003[7]. Like other malaria endemic countries, rapid and accurate malaria diagnosis is critical for malaria control in remotes areas such as the Amazonian region. In areas where microscopy services are not available, diagnosis relies on blood films sent to distant centers for evaluation, with results taking up to 3 weeks to return [8]. Presumptive treatment base on clinical symptoms is common in these areas with a sensitivity as low as 39% [9].

Generally, HRP2-detecting RDTs show comparable sensitivity to quality microscopy, their performance, however, has been reported to be variable in different settings [10]–[18] including Peru. One study carried out in the Peruvian Amazon during 1998 and 1999 using ParaSight F™ commercial kit, which targets HRP2 antigen, showed 95% of sensitivity [19], [20]. However, other trials conducted in subsequent years using other RDTs that target HRP2, including ParaScreen™[21] and ICT MALARIA *pf*/*pv* (AMRAD™) [22], showed a sensitivity below 75%.

The performance of RDTs in the field is influenced by many factors including quality of manufacture, storage conditions [23], ability of the user to correctly interpret the result [24], [25] and parasite density [26]. For RDTs detecting HRP2, genetic diversity of the *Pfhrp2* gene and the protein could also affect their performance. Indeed, the deduced amino acid sequences of HRP2 of different isolates are highly polymorphic [27], whereby the number and sequence of specific repeats present in the PfHRP2 proteins varies widely. Moreover the epitopes recognised by the Monoclonal antibodies (MAbs) specific for HRP that are available for and used in RDTs also vary significantly between parasites [28]. While available evidence indicates that HRP2 is the major target detected in HRP2 RDTs, HRP3 is also secreted and can be detected by MAbs raised against HRP2 [28]. The role of HRP3 in performance of HRP diagnostic tests is not well defined.

Another possible factor affecting the sensitivity of HRP2-detecting RDTs is failure of the parasite to express the antigen, due to deletion of the gene or frame shift mutations, or alterations in protein expression. However, to date parasite lines lacking either or both *pfhrp2* or *pfhrp3* genes have only been identified following laboratory adaptation to *in vitro* culture [29], [30], and in the progeny of a genetic cross [31], No reports exist in the literature of parasites collected from human patients lacking one or both of these genes.

During our recent characterization of *pfhrp2* in parasite isolates collected from several countries as part of the World Health Organisation (WHO) – Foundation for Innovative New Diagnostics (FIND) malaria RDT evaluation program [32], we identified a number of *P. falciparum* samples collected in Iquitos, Peru, that were positive by microscopy but tested negative with HRP2-detecting RDTs. Further characterisation of these samples revealed that they lacked either *pfhrp2* or *pfhrp3* or both. Following these findings, we conducted a retrospective study to determine the prevalence of *pfhrp2/3* gene deletion in *P. falciparum* samples collected from different malaria endemic areas in Peru. In this paper we report the genetic characterization and the geographic distribution of the parasites lacking *pfhrp2* and/or *pfhrp3* in the Peruvian Amazon region. The implications of these findings for various countries in South America that are moving to introduce malaria RDTs under programmes supported by the Global Fund to fight AIDS, Tuberculosis and Malaria (GFATM) are discussed.

## Methods

### Study Sites and Sample Collection

**Figure 1:**
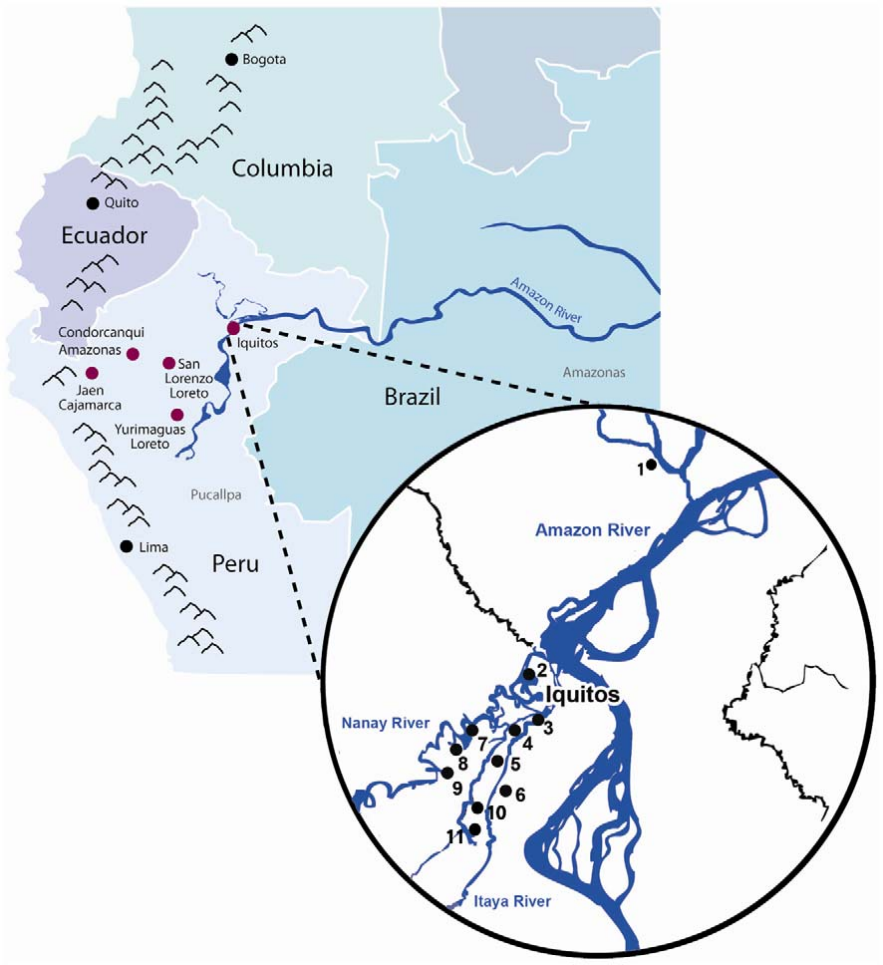
Map of Iquitos and the Peruvian Amazon. The locations of health centers in Iquitos where the *P. falciparum* samples were collected are shown in solid circles with numbers: 1, Mazan; 2, Moronacocha; 3, Cardozo; 4, San Juan de Miraflores; 5, 9 de octubre; 6, El progreso; 7, Santa Clara de Nanay; 8, Santo Tomas; 9, Zungarococha; 10, Moralillo; 11, Varillal. The locations where samples were collected in other Peruvian Amazon areas are shown as red dots.

The samples used in this study came from two sources:

1. An active case detection survey conducted in 2007 in communities around Iquitos, the main city in the Peruvian Amazon area (between Nanay river, Itaya river and the left margin of the Amazon river), Peru (Figure 1) as part of the WHO –FIND malaria RDT evaluation program. Finger prick blood was collected from 16 *P. falciparum* patients onto filter paper and dried. Of these 9 samples met the inclusion criteria set for the RDT QA program (HIV-hepatitis B/C free, with no history of antimalarial chemotherapy in the last month, and parasitemia >2000 parasites/µL). Blood spots were transported to the Australian Army Malaria Institute for characterisation. Aliquots of frozen blood samples were sent to the malaria specimen bank at the Centers for Disease Control and Prevention (CDC), Atlanta, USA for species confirmation by 18s rRNA nested PCR and archiving, and to the Hospital for Tropical Disease (HTD), London, UK, for ELISA. The collection, transport and storage of the blood samples were approved by the Human Ethics Committee from Universidad Peruana Cayetano Heredia (UPCH 52707), Peru, while the testing of blood samples for *pfhrp2* diversity was approved by the Australian Defence Human Research Ethics Committee (ADHREC 377/05). The remaining 7 samples were characterised together with the retrospective samples.
2. Samples collected during previous malaria studies from different areas of the Peruvian Amazon. A total of 159 *P. falciparum* samples, collected in different studies conducted between 2003 and 2007 by the Laboratory of Malaria at the Institute of Tropical Medicine “Alexander von Humboldt”-UPCH, were available for testing. 114/159 samples came from three different projects carried out at 11 Health post centers in peri-urban areas around Iquitos city (Table 1). The location and distance of these health centers to Iquitos city are: Cardozo (3.5 Km), Mazan (38 Km), Moronacocha (2 Km), San Juan de Miraflores (4.6 Km), 9 de octubre (2 Km) El progreso (2.5 Km), Santa Clara de Nanay (12 Km), Santo Tomas (16 Km), Moralillo (15 Km), Varillal (20 Km) and Zungarococha (15 Km) (Figure 1).

**Table 1:** Origin of the *P. falciparum* samples used.

*Study area (Project)*	*Collection Year*	*Number of samples*
Iquitos (ARTEKIN)	2003–2005	89
Iquitos (PAMAFRO)	2006	12
Iquitos (RDT QA)	2007	16*
Condorcanqui	2006–2008	8
Jaen	2005	2
San Lorenzo	2007	17
Yurimaguas	2004	15
Total *P. falciparum* positive samples		159

Note: All samples were diagnosed by PCR and microscopy, except for the area of San Lorenzo where RDT (OptiMAL™) was used.

* Nine samples described in the Table 2 are included in this group.

The remaining 34 samples came from other areas in the Peruvian jungle as well as from different projects (Table 1). From this group, 12 samples were collected in Yurimaguas, located between the Marañon and Paranapura rivers (445 Km south of Iquitos); 15 samples were from San Lorenzo, located on the shores of the Huallaga river and nine hours from Yurimaguas by boat (110 Km south-west from Iquitos); 7 samples came from the area called High Jungle 2 from Jaen located 620 Km west of Iquitos and 5 samples from Condorcanqui located 524 Km west from Iquitos (Figure 1). The Universidad Peruana Cayetano Heredia Ethical Committee approved the use of parasite DNA for this study.

### Other Parasite Isolates and Laboratory Lines

Genomic DNA from 10 *P. falciparum* field isolates collected as part of the same WHO program: Cambodia (n = 3), Myanmar (n = 2), Nigeria (n = 2), Tanzania (n = 2) and Madagascar (n = 1), as well as from 4 laboratory lines (3D7, 7G8, Dd2 and D10) was used for PCR amplification of genes immediately flanking *pfhrp2* and *pfhrp3*.

### Extraction of Parasite DNA

Genomic DNA was isolated from dried blood spots on filter paper, frozen blood or cryopreserved laboratory cultures using the QIAblood Mini™, Quelex™-100 or Qiagen™ kits (QIAGEN) in accordance with the manufacturer's instructions. Phenol-Chloroform method was used to extract parasite DNA from nitrocellulose strips of used RDTs [33].

### Confirmation of *P. falciparum* Infection by PCR

*P. falciparum* infections were confirmed by PCR amplification of 4 *P. falciparum* specific genes:*18sRNA gene, pfmsp1, pfmsp2* and *pfglurp*. The *P. falciparum* 18sRNA gene was amplified using methods described by Padley *et al* [34], modified by shortening the extension time to 50 sec and extending the number of cycles to 45, or as described by Rubio *et al* [35]. The amplification *pfmsp1*, *pfmsp2* and *pfglurp* was performed using the nested primers described elsewhere [36] for 45 cycles.

### Detection of the *pfhrp2* and *pfhrp3* Genes by PCR

The amplification of *pfhrp2* and *pfhrp3* exon 2 was performed using primers and conditions as described elsewhere [27] for 45 cycles. For the 9 samples collected in 2007, three further PCR protocols were undertaken: to amplify across exons 1 and 2 of both *pfhrp2* and *pfhrp3* and across exon 1 and the entire exon 2 of *pfhrp3* using a second set of primers (Table S1).

### Detection of Genes Flanking *pfhrp2* and *pfhrp3* by PCR

Primers (Table S1) were designed to amplify genes immediately flanking *pfhrp2*: MAL7P1.230 (5.535 kb upstream) and MAL7P1.228 (6.49 kb downstream), and those immediately flanking *pfhrp3*: MAL13P1.485 (4.404 kb upstream) and MAL13P1.475 (1.684 kb downstream). The PCR conditions and expected product sizes are described in Table S1.

### DNA Sequencing

PCR products were purified by the use of spin columns (Roche), and used in a standard dye-terminator DNA sequencing reaction (ABI).

### Detection of HRP2 Protein Using ELISA

The quantity of HRP2 protein in the 9 samples collected in 2007 was measured using the Malaria Ag CELISA kit (CELLABS Pty, Sydney, Australia) following manufacturer's instructions. The protein was quantified by plotting absorbance values against a standard curve of recombinant HRP2. Protein levels for parasite pLDH (Standard Diagnostics, Kyonggi-do, Korea) and aldolase (Centers for Disease Control, Atlanta, USA) were also measured by ELISA with recombinant protein standard curves [37].

## Results

### Eight of the 9 *P. falciparum* Samples Collected during the 2007 Survey in Iquitos Lack *pfhrp2* and *pfhrp3*

As part of the WHO – FIND malaria RDT evaluation program [32] we undertook characterization of *pfhrp2* in 9 *P. falciparum* patient samples collected in Iquitos, Peru. Eight of the nine samples did not yield the expected PCR product using our standard primers spanning the full length of *pfhrp2* exon 2, or in subsequent experiments using primers spanning a shorter sequence of exon 1 and exon 2 (Table 2, Figure S1, S2), indicating that these parasites lacked the *pfhrp2* gene. In further testing 4 of these 9 samples also tested negative for the *pfhrp3* gene by PCR specific for exon 2 of this gene. Among the 5 samples that had yielded the expected PCR products for exon 2 of *pfhrp3* two (PE01F07 and PE01F11) failed to yield a product with primers spanning exon 1 and exon 2, or exon 1 and full length exon 2 of *pfhrp3* (Table 2, Figure S2) indicating a truncation of exon 1.

**Table 2:** Gene amplification and protein detection results for the 9 samples collected in 2007 (positive and negative results are represented by + and − respectively).

Isolates	Multiplex rRNA	Allelic Type *pfmsp1*	Allelic Type *pfmsp2*	Allelic type *pfglurp*	MAL7 P1.230 -5.535 kb	hrp2- exon 1–2	hrp2- exon 2	MAL7 P1.228 +6.49 kb	MAL13 P1.485 -4.404 kb	hrp3-exon 1–2	hrp3 exon 1–2 FL	hrp3- exon 2	MAL13 P1.475 +1.684 kb	ELISA HRP2 (ng/mL)	ELISA pLDH (ng/mL)
PE01F04*	Pf	A	1	I	+	+	+	+	+	+	+	+	+	235	503.4
PE01F06	Pf	A	1	I	−	−	−	+	+	+	+	+	+	0	19.27#
PE01F07	Pf	B	2	II	−	−	−	+	+	−	−	+	−	0	3534.75
PE01F011*	Pf	A	2	III	−	−	−	+	+	−	−	+	−	0	698.25
PE01F015	Pf	A	2	IV	−	−	−	+	+	+	+	+	+	0	41.85
PE01F016*	Pf	A	2	IV	−	−	−	+	−	−	−	−	−	0	38.65
PE01F017	Pf	A	2	IV	−	−	−	+	−	−	−	−	+	0	467.1
PE01F018*	Pf	A	2	IV	−	−	−	+	−	−	−	−	−	0	117.7
PE01F019*	Pf	A	2	IV	−	−	−	+	−	−	−	−	−	0	61.3

* DNA isolated from frozen blood;

#Measured at a dilution of 200 parasite/µL.

### Confirmation that the Parasite Isolates Were *P. falciparum* Infections and Investigation for Genetic Diversity of the Parasites

To verify the malaria parasite species and to investigate genetic diversity in the parasites, a multiplex PCR reaction that amplifies the 18s rRNA gene of 4 *Plasmodium* species commonly infecting humans, and three polymorphic *P. falciparum* genes *pfmsp1*, *pfmsp2 and pfglurp* was undertaken. Results of these experiments confirmed that all 9 samples were *P. falciparum*, with no evidence of contamination or co-infection with another species (Table 2, Figure S2). Of note, the sizes of the PCR products of the polymorphic genes *pfmsp1, pfmsp2* and *pfglurp* were observed to vary, revealing 2, 2 and 4 allelic types at these loci respectively and establishing that the isolates were not clonal in nature. The combined three-loci-type separated the isolates into four different haplotypes: 1) PE01F04 and PE0F06, 2) PE01F07, 3) PE01F11 and 4) PE01F15 to PE01F19 (Table 2).

### Investigation of the Genetic Basis of These Deletions

To investigate for larger chromosomal deletions encompassing the two *hrp* genes, PCR reactions were undertaken to amplify the two genes flanking each of *pfhrp2* and *pfhrp3*. The specificity of these PCR reactions was first tested on well characterized laboratory lines of *P. falciparum*. PCR products of the expected size were amplified from two laboratory lines, 3D7 and 7G8, and the identity of the 3D7 PCR product confirmed by DNA sequence analysis. PCR amplification for *pfhrp2* in Dd2, a laboratory line known to lack *pfhrp2* failed to amplify *pfhrp2* and both flanking genes; appropriate PCR products for *pfhrp3* and both flanking genes were amplified from this line. PCR reactions undertaken with D10, another laboratory line known to lack *pfhrp2*, failed to amplify *pfhrp2* and the upstream gene, but a PCR product of the expected size was present for the downstream gene, MAL7P1.228. Likewise, PCR amplification of *pfhrp3* and its flanking genes was successful for D10. PCR reactions were then undertaken with 10 field isolates from different geographic areas that had yielded the expected *pfhrp2* and *pfhrp3* products, and also yielded products of the expected size for the four genes flanking both *pfhrp2* and *pfhrp3* (Table S2).

When the Peruvian isolate PE01F04, the only isolate positive for *pfhrp2* in this subset, was subject to PCR for the genes flanking *pfhrp2*, it yielded products for both flanking genes as was observed with 3D7 and 7G8. PCR reactions undertaken with the remaining 8 Peruvian samples, which were negative for *pfhrp2*, resulted in amplification of the expected product for the downstream gene but failed to amplify the gene upstream of *pfhrp2*, in a pattern resembling that observed with D10. Isolates PE01F04, PE01F06 and PE01F15 that had yielded *phfhp3* PCR products were also PCR-positive for both genes flanking *pfhrp3*. PE01F07 and PE01F11, isolates where evidence had indicated that may have truncated exon 1 were PCR positive for the upstream gene but negative for the downstream gene. The four samples that had failed to yield a PCR product for *pfhrp3* were all PCR-negative for the upstream flanking gene, and 3 of the 4 samples were also negative for the downstream gene (Table 2, Figure S2).

### Verification that These Parasites Do Not Produce HRP Protein

To investigate whether these parasites lacked histidine rich protein all 9 samples were screened for HRP2 protein levels by a HRP antigen capture ELISA. Only one of the 9 samples (PE01F04) had detectable histidine rich protein 2, giving an estimated 235 ng/mL protein (Table 2). In contrast, all 9 samples had measurable levels of pLDH and aldolase. When tested on 3 different commercially available HRP2-detecting RDTs, PE01F04 again was the only sample test positive with all 3 RDTs. Two samples lacking *pfhrp2* but having intact *pfhrp3* (PE01F06 and PE01F15) tested weakly positive on the ICT combo kit (Cape Town, South Africa). PE01F06 tested weakly positive but PE01F15 was negative with 2 other RDT brands (CareStart Malaria HRP2 and CareStart Malaria Combo. AccessBio, Monmouth, NJ, USA). The remaining 5 samples lacking both *phfrp2* and *pfhrp3* were negative on all 3 different RDTs.

### A High Proportion of Retrospectively Collected Samples from the Amazon Region of Peru Lack *pfhrp2* and *pfhrp3* Genes

We then undertook study of the presence of *hrp*-deleted parasites from other areas of Peru. PCR amplifications of the *18SrDNA* gene and *pfglurp* was performed for 159 samples that had been collected in previous studies, of which 148 were positive for both markers confirming *P. falciparum* infection and indicating a good quality of DNA. The size of the PCR product for *pfglurp* varied ranging from 550 to 900 bp, indicating a total of 6 genotypes (Table 3). These 148 samples were then tested for the presence of *pfhrp2* and *pfhrp3* by PCR.

**Table 3:** *pfglurp* allelic type detected in the retrospective samples.

Genotype	Size in base pairs	Number of samples
A	550–600	27
B	600–650	25
C	650–700	19
D	700–750	36
E	750–800	29
F	800–900	12
Total samples		148

Of 114 samples collected from peri-urban areas of Iquitos, PCR for the *pfhrp2* and the *pfhrp3* genes failed in 36% and 67% of the samples respectively (Table 4), despite being positive for *18sRNA* and *pfglurp*. The proportion of samples failing to amplify both *pfhrp2* and *pfhrp3* was 18.4% (21/114), while the proportion of samples positive for both genes was only 15.78% (18/114).

**Table 4:** Proportion of *P. falciparum* isolates positive or negative for *pfhrp2/3* genes in different areas of the Peruvian Amazon.

Area	No.	*pfhrp2* – PCR		*pfhrp3* – PCR		*pfhrp2/pfhrp3*	
		Pos (%)	*Neg* (%)	Pos (%)	*Neg (%)*	*double positive (%)*	*double negative (%)*
Iquitos	114	73 (64)	41 (36)	35 (31)	79 (69)	18 (15.8)	21 (18.4)
Condorcanqui	5	0 (0)	5 (100)	4 (80)	1 (20)	0 (0)	1 (20)
San Lorenzo	15	8 (53)	7 (47)	2 (13)	13 (87)	1 (6.7)	6 (40)
Yurimaguas	12	6 (50)	6 (50)	2 (17)	10 (83)	1 (8.3)	4 (33.3)
Jaén	2	0 (0)	2 (100)	2 (100)	0 (0)	0 (0)	0 (0)
Combined	148	87 (59)	**61 (41)**	45 (30)	**103 (70)**	20 (13.5)	32 **(21.6)**

### *pfhrp2* and *pfhrp3* Deleted Parasites are Widely Distributed in the Peruvian Amazon

Parasites lacking *pfhrp2* and *pfhrp3* were also identified in other areas in the Peruvian jungle. The proportion of *pfhrp2* and *pfhrp3* positive and negative in samples collected in Condorcanqui, San Lorenzo, Yurimaguas and Jaen is shown in table 4. The proportion varied between parasites collected in different areas. While the number of samples collected in these areas was small, overall a high proportion of parasites negative for *pfhrp2* and *pfhrp3* was observed in these areas and was comparable to levels observed around Iquitos. When all areas are combined, we observed that a total of 41% and 70% of the samples were negative to *pfhrp2* and *pfhrp3*, respectively (Table 4). The percentage of samples that were negative for both *pfhrp2* and *pfhrp3* was 21.6% (32/148), while the percentage of samples that were positives for both *pfhrp2* and *pfhrp3* was 13.5% (20/148).

### Amplification Stringency

In order to rule out the possibility that failure to PCR amplify the *pfhrp2/3* genes was due to possible polymorphisms at the primer binding sites, an additional PCR reaction was undertaken for samples that were negative for either genes using the same primers with lower annealing temperatures (down to 42°C). All samples and the negative controls remained negative, while the positive control (NF54) was positive, suggesting that no small mismatches occurred at the primer sites (Figure S3).

## Discussion

Laboratory lines of *P. falciparum* that lack *pfhrp2* or *pfhrp3* or both genes have been reported following adaptation to and long term growth under *in vitro* culture conditions [29]–[31]. However, this is the first report of clinical isolates of parasites taken directly from infected human subjects in endemic areas lacking these genes. As part of the WHO-FIND malaria RDT evaluation program we have examined *pfhrp2* sequence variation of over 500 clinical parasite samples collected from many different malaria endemic areas in Africa, South East Asia, West Pacific and South America ([27] and Baker et al unpublished) and have not encountered *P. falciparum* samples lacking *pfhrp2* and/or *pfhrp3* elsewhere. In Peru, we identified, for the first time, not only parasites from clinical patients lacking these hrp genes but also a wide spread pattern of parasites lacking these genes in the Peruvian Amazon with an overall frequency of 41% and 70% of parasites without *pfhrp2* and *pfhrp3*, respectively and 21.6% for double deletions.

### *pfhrp2* and/or *pfhrp3* PCR Negative Samples Lack These Genes Due to Chromosomal Deletions

A plausible hypothesis for the negative PCR results for *pfhrp2* and/or *pfhrp3* is that these parasites lack these genes as a consequence of chromosomal deletion. A commonly used method for characterizing genetic rearrangements and deletions is a genomic hybridization where parasite genomic DNA or parasite chromosomes are hybridised with the target gene (generally by Southern Blot), in this case, *pfhrp2* or *pfhrp3*. Since we only had blood spots on filter paper and small aliquots of frozen whole blood, there was insufficient parasite genomic DNA to use this method. Instead, we performed a number of independent PCR experiments to characterise the samples to obtain supporting evidence that the parasites actually lack these genes. These experiments also ensured that the negative PCR reactions for *pfhrp2* and *pfhrp3* were not due to poor quality or insufficient parasite DNA. To rule out the possibility of sequence polymorphism at primer binding sites, further PCR experiments were undertaken using alternative primers designed to anneal at highly conserved sequence stretches in both *pfhrp2* and *pfhrp3*. All isolates that were PCR negative using the exon 2 *pfhrp2* and *pfhrp3* primers were also PCR negative using these latter primers.

Deletions of *pfhrp2* and *pfhrp3* previously reported in laboratory lines have all been shown to result from chromosomal deletions, where a large fragment of a chromosome usually containing several genes is deleted [29]–[31]. Further evidence that 8 of the 9 and 6 out 9 Iquitos parasites lack *pfhrp2* or both *pfhrp2* and *pfhrp3*, respectively, is provided by the observation that the negative PCR reactions for the flanking genes were consistent with those for *pfhrp2* and *pfhrp3*. Taken together, the results suggest that a fragment on chromosome 7 that contains *pfhrp2* and its immediate upstream gene (MAL7P1.230) encoding a hypothetical protein has been deleted in these parasites. In all 8 isolates negative for *pfhrp2*, the gene immediately downstream from *pfhrp2* encoding HSP70 is preserved. On chromosome 13, PCR reactions for both *pfhrp3* and the genes immediately upstream (MAL13P1.485), and in most cases for the downstream gene (MAL13P1.475) were negative for 4 of the 9 samples, indicating a chromosomal deletion. This finding is supported by independent data showing that chromosomal deletions in both the region on chromosome 7 where *pfhrp2* is located, and the region on chromosome 13 where *pfhrp3* is located, are possible and have been observed in some laboratory parasite lines [38].

### Parasite Lack *pfhrp2* and/or *pfhrp3* Do Not Express HRP2 Protein and Are Not Detectable by HRP2 Detecting RDTs

Most importantly from the standpoint of diagnostic assay performance, the final evidence of gene deletion or inactivation, came from the assay for histidine-rich protein by ELISA where results show that with the exception of one parasite isolate none have detectable histidine rich protein 2, though all isolates have detectable pLDH. The single isolate in this subset of samples in which histidine-rich protein could be detected was positive for both *pfhrp2* and *pfhrp3*. When tested using HRP2-detecting RDTs, parasites having double gene deletions all tested negative on different HRP2-detecting RDTs demonstrating the lack of antigens in these parasites. Parasites having deleted only *phfrp2* but not *pfhrp3* were detectable by some RDTs but not others resembling the ELISA results due, most likely, to different antibodies used in different RDTs or ELISA test which recognise different epitopes that may or may not be shared between *pfhrp2* and *pfhrp3* [28].

### A Large Proportion of P. falciparum Parasites in Peruvian Amazon Lack pfhrp2 and pfhrp3

Evidence for a widespread distribution of HRP-deleted parasites across the Peruvian Amazon comes from our observation that a large proportion of parasites lacking *pfhrp2* and or *pfhrp3* were observed in other areas from the Peruvian jungle between 110 and 620 kilometres from Iquitos (Condorcanqui, San Lorenzo, Yurimaguas and Jaen) and collected over a period of 4 years. These findings indicate that the *hrp*-negative parasites are not restricted to a specific area, but are distributed in much of the malaria endemic areas in Peru, and raises the possibility that such parasites may be present in neigbouring countries with identical patterns of malaria and sharing the same river system for transport.

### The Absence of HRP2 and HRP3 Likely Explains the Poor Performance of Some HRP2-Detecting RDTs Previously Reported in the Peruvian Amazon

The previously reported sensitivity for these tests ranged from 70% [21] to 65% (MALARIA *pf*/*pv* (AMRAD™)) [22], consistent with our findings, while *pfLDH*-detecting RDTs (OptiMAL) have shown higher sensitivity in the Peruvian Amazon [39]–[42]. ParaSight F (Becton-Dickensen, USA) was trialed in Iquitos for several years before 2000 showing good sensitivity in detecting *P. falciparum* infections [19], [20]. It is possible that at that time, parasites lacking *pfhrp2* were not present in high proportions to affect the performance of this test. Therefore, the widespread of parasites lacking *pfhrp2* gene may be a relatively recent event.

### Parasite Evolution

The evolutionary process that produced these parasites is unknown but intriguing. It is notable that the proportion of parasites lacking *pfhpr3* is higher than those lacking *pfhrp2* in samples collected from almost all locations, while the proportion of parasites lacking both genes is 21.6%. This suggests that parasites lacking *pfhrp3* may have been present in Peru longer than those lacking *pfhrp2*. It is possible that parasites lacking *pfhrp3* had undergone a genetic cross with parasites lacking *pfhrp2* and produced progeny lacking both genes. As four different *pfmsp1/pfmsp2/pfglurp* haplotypes were observed in the subset of 9 isolates lacing *pfhrp2*, chromosomal deletions on chromosome 7 may have occurred frequently in the area. A similar genetic cross event and outcome has been reported in a laboratory cross where Dd2 (*pfhrp2* negative) and HB3 (*pfhrp3* negative) were crossed. While most progeny inherited either a *pfhrp2* or a *pfhrp3* deletion, one progeny (3BD5) lacked both *pfhrp2* and *pfhrp3* according to genetic linkage analysis [31].

While the function(s) of *pfhrp2* and *pfhrp3* is unclear, they do not appear to be essential for *in vitro* growth because progeny lacking either gene or both *pfhrp2* and *pfhrp3* remain viable *in vitro*, with exhibition of a rapid growth phenotype. This observation of wild-type parasites lacking both *pfhrp2* and *pfhrp3* from patients in Peru indicate that these parasites are viable *in vivo*. Further study of parasite virulence and fitness in these deletion mutants would be of interest, but they are obviously capable of transmission and of causing disease as all cases were symptomatic. Certainly, the mechanism of selection that has driven these parasite genotypes to be common in this Peruvian population of *P. falciparum* remains to be determined.

### HRP2-Detecting RDTs Are Not Reliable in Peru

The most important implication of this finding is that, in most malaria endemic areas of Peru, diagnosis of the malaria due to *P. falciparum*, the species responsible for almost all malaria mortality and much of the morbidity, would commonly fail if HRP2-detecting RDTs are used. This raises a serious question concerning on the value of the use of HRP2-detecting RDTs in Peru. Because *P. falciparum* is endemic in a great area of the Peruvian Amazon with 98% of the *falciparum* malaria cases in Peru occur in this area [6], any malaria RDT used here must detect *P. falciparum* with high sensitivity. The high proportion of samples in which *pfhrp2* could not be amplified (41%) and high proportion of samples lacked both *pfhrp2* and *pfhrp3* (21.6%) in this study, which were collected from different areas and at different time points, clearly demonstrate that HRP2-detecting RDTs have limited reliability for detecting *P. falciparum* in Peru. RDTs targeting other antigens (pLDH and aldolase) and quality microscopy should be used for malaria diagnosis.

As Peru borders on several countries that share the Amazon River basin and where malaria transmission occurs without respect for national borders, it is unlikely that parasites lacking *pfhrp2* and *pfhrp3* are confined to Peru. It is therefore important that investigations be performed in other areas in South America urgently where *P. falciparum* is endemic to determine the presence and geographical spread of parasites lacking the *pfhrp2* and *pfhrp3* genes before the large scale implementation of malaria RDTs in this area. Investigations should also be carried out to monitor the presence and spread of parasites with gene deletions in areas outside of South America to ensure the best performance of malaria RDTs globally.

## Supporting Information

Table S1 Primer sequences, PCR conditions and expected product sizes
(0.01 MB DOC)

Table S2 Amplification of pfhrp2, pfhrp3 and their immediate flanking genes in laboratory lines and field isolates.
(0.01 MB DOC)

Figure S1 Schematic illustration of pfhrp2 and pfhrp3 gene structures and primer binding sites. Filled arrows represent primers that amplify the full length exon 2; open arrows representing primers amplify across exon 1 and exon 2.
(0.05 MB TIF)

Figure S2 PCR products visualised on agarose gels. Panels a) to l) shows the PCR results for: a) pfhrp2 exon 2, b) pfhrp2 exon 1-exon 2, c) pfhrp3 exon 2, d) pfhrp3 exon 1-exon 2, e) 18s rRNA, f) pfglurp, g) pfmsp1, h) pfmsp2, i) MAL7P1.228, j) MAL7P1.230, k) MAL13P1.475, l) MAL13P1.480. Numbers 1 to 12 represent lanes on each gel: 1: Marker; 2: PE01 F04; 3: PE01 F06; 4: PE01 F07; 5: PE01 F11; 6: PE01 F15; 7: PE01 F16; 8: PE01 F17; 9: PE01 F18; 10: PE01 F19; 11: 3D7; 12: No DNA
(0.51 MB TIF)

Figure S3 Temperature Gradient for PCR. A: pfhrp2 positive samples. Lane 1: 100 pb plus Molecular marker (MM) - RocheTM; Lane 2: 42.0°C; Lane 3: 43.0°C; Lane 4: 45.0°C; Lane 5: 47.0°C; Lane 6: 48.0°C; Lane 7: 50.0°C; Lane 8: NF54; Lane 9: Negative control (NC) to 50.0°C. B: pfhrp2 negative samples. Lane 1: MM; Lane 2: 50.0°C; Lane 3: 48°C; Lane 4: 47.0°C; Lane 5: 45.0°C; Lane 6: 44.0°C; Lane 7: 43.0°C; Lane 8: 42.0°C; Lane 9: NF54 (50.0°C); Lane 10: NC. C: pfhrp3 positive samples. Lane 1: MM; Lane 2: 50.0°C; Lane 3: 48.0°C; Lane 4: 47.0°C; Lane 5: 45.0°C; Lane 6: 44.0°C; Lane 7: 43.0°C; Lane 8: 42.0°C; Lane 9: NC. D: pfhrp3 negative samples. Lane 1: MM; Lane 2: 50.0°C; Lane 3: 48.0°C; Lane 4: 47.0°C; Lane 5: 45.0°C; Lane 6: 44.0°C; Lane 7: 43.0°C; Lane 8: 42.0°C; Lane 9: 41.0°C; Lane 10: 40.0°C; Lane 11: NC.
(0.29 MB TIF)
