# Supplementary material for: A Large Proportion of P. falciparum Isolates in the Amazon Region of Peru Lack pfhrp2 and pfhrp3: Implications for Malaria Rapid Diagnostic Tests
Source: PLoS One. 2010 Jan 25;5(1):e8091. doi: 10.1371/journal.pone.0008091 (PMC2810332; doi:10.1371/journal.pone.0008091)
Supplement: Table S1 — Primer sequences, PCR conditions and expected product sizes (0.01 MB DOC) [file pone.0008091.s001.doc]

Table S1. Primer sequences, PCR conditions and expected product sizes

| Gene name | Primer sequence | PCR conditions | Expected product size |
| --- | --- | --- | --- |
| MAL7P1_228 | 5' AGA CAA GCT ACC AAA GAT GCA GGT G 3' | 94ºC for 10 min, followed by 94ºC for 30 sec, 60ºC for 30 sec, 68ºC for 1 min | 227 bp |
|  | 5' TAA ATG TGT ATC TCC TGA GGT AGC 3' |  |  |
| MAL7P1_230 | 5' TAT GAA CGC AAT TTA AGT GAG GCA G 3' | As above except for the annealing temperature at 68ºC | 346 bp |
|  | 5' TAT CCA ATC CTT CCT TTG CAA CAC C 3' |  |  |
| MAL13P1_475 | 5' TTC ATG AGT AGA TGT CCT AGG AG 3' | As above except for the annealing temperature at 55ºC | 260 bp |
|  | 5' TCG TAC AAT TCA TCA TAC TCA CC 3' |  |  |
| MAL12P1_485 | 5' TTG AGT GCA ATG ATG AGT GGA G 3' | As above except for the annealing temperature at 60ºC | 287 bp |
|  | 5' AAA TCA TTT CCT TTT ACA CTA GTG C 3' |  |  |
| *Pfhrp2* exon 1-2 | 5’ TAT CCG CTG CCG TTT TTG CC 3’ | As above except for the annealing temperature at 57^o^C | 303 bp |
|  | 5’ AGC ATG ATG GGC ATC ATC CTA 3’ |  |  |
| *Pfhrp3* exon 1-2 | 5’ TAT CCG CTG CCG TTT TTG CTT CC 3’ | As above | 303 bp |
|  | 5’ TGC ATG ATG GGC ATC ACC TG 3’ |  |  |
