# Supplementary material for: A Large Proportion of P. falciparum Isolates in the Amazon Region of Peru Lack pfhrp2 and pfhrp3: Implications for Malaria Rapid Diagnostic Tests
Source: PLoS One. 2010 Jan 25;5(1):e8091. doi: 10.1371/journal.pone.0008091 (PMC2810332; doi:10.1371/journal.pone.0008091)
Supplement: Table S2 — Amplification of pfhrp2, pfhrp3 and their immediate flanking genes in laboratory lines and field isolates. (0.01 MB DOC) [file pone.0008091.s002.doc]

Table S2. Amplification of *pfhrp2*, *pfhrp3* and their immediate flanking genes in laboratory lines and field isolates.

| Lines/  Isolates | MAL7P1.230 | hrp2- exon 2 | MAL7P1.228 | MAL13P1.475 | hrp3- exon 2 | MAL13P1.485 |
| --- | --- | --- | --- | --- | --- | --- |
| 3D7 | + | + | + | + | + | + |
| 7G8 | + | + | + | + | + | + |
| Dd2 | - | - | - | + | + | + |
| D10 | - | - | + | + | + | + |
| MY7F01 | + | + | + | + | + | + |
| MY7F02 | + | + | + | + | + | + |
| CAM028 | + | + | + | + | + | + |
| CAM037 | + | + | + | + | + | + |
| NL1F14 | + | + | + | + | + | + |
| NL1F15 | + | + | + | + | + | + |
| MG01F19 | + | + | + | + | + | + |
| TZ1F8 | + | + | + | + | + | + |
| TZ1F11 | + | + | + | + | + | + |
| C3F6 | + | + | + | + | + | + |
