# Supplementary figures and images for: A Large Proportion of P. falciparum Isolates in the Amazon Region of Peru Lack pfhrp2 and pfhrp3: Implications for Malaria Rapid Diagnostic Tests
Source: PLoS One. 2010 Jan 25;5(1):e8091. doi: 10.1371/journal.pone.0008091 (PMC2810332; doi:10.1371/journal.pone.0008091)

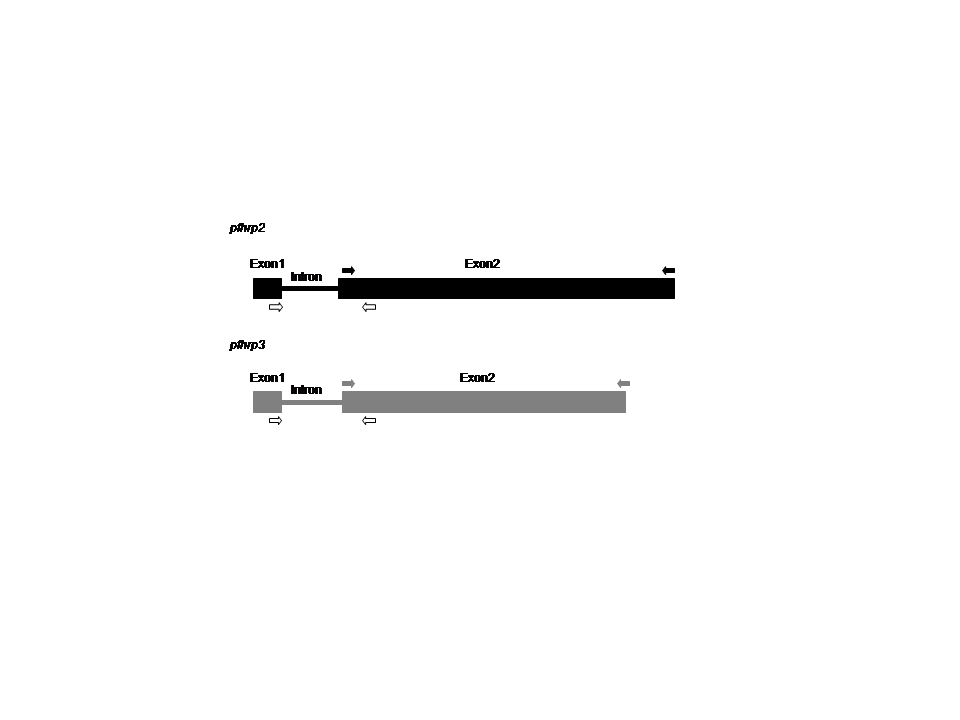

Supplement: Figure S1 — Schematic illustration of pfhrp2 and pfhrp3 gene structures and primer binding sites. Filled arrows represent primers that amplify the full length exon 2; open arrows representing primers amplify across exon 1 and exon 2. (0.05 MB TIF) [file pone.0008091.s003.tif]

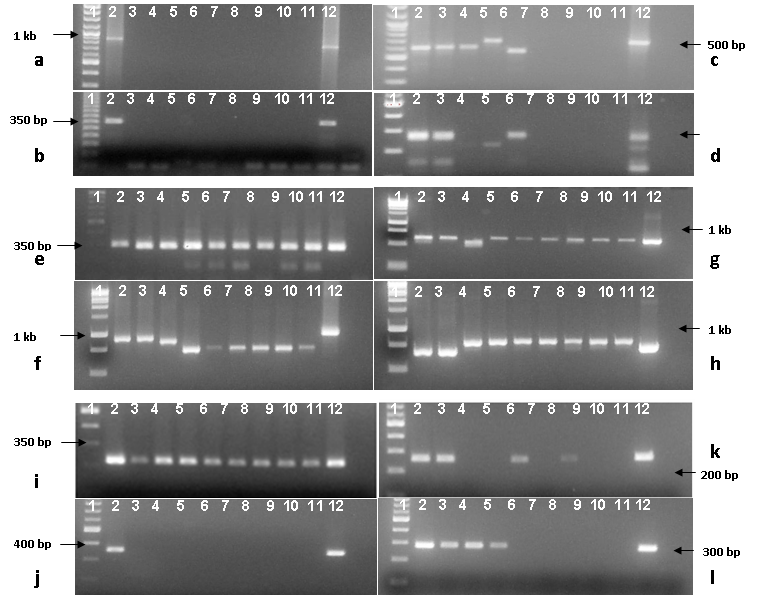

Supplement: Figure S2 — PCR products visualised on agarose gels. Panels a) to l) shows the PCR results for: a) pfhrp2 exon 2, b) pfhrp2 exon 1-exon 2, c) pfhrp3 exon 2, d) pfhrp3 exon 1-exon 2, e) 18s rRNA, f) pfglurp, g) pfmsp1, h) pfmsp2, i) MAL7P1.228, j) MAL7P1.230, k) MAL13P1.475, l) MAL13P1.480. Numbers 1 to 12 represent lanes on each gel: 1: Marker; 2: PE01 F04; 3: PE01 F06; 4: PE01 F07; 5: PE01 F11; 6: PE01 F15; 7: PE01 F16; 8: PE01 F17; 9: PE01 F18; 10: PE01 F19; 11: 3D7; 12: No DNA (0.51 MB TIF) [file pone.0008091.s004.tif]

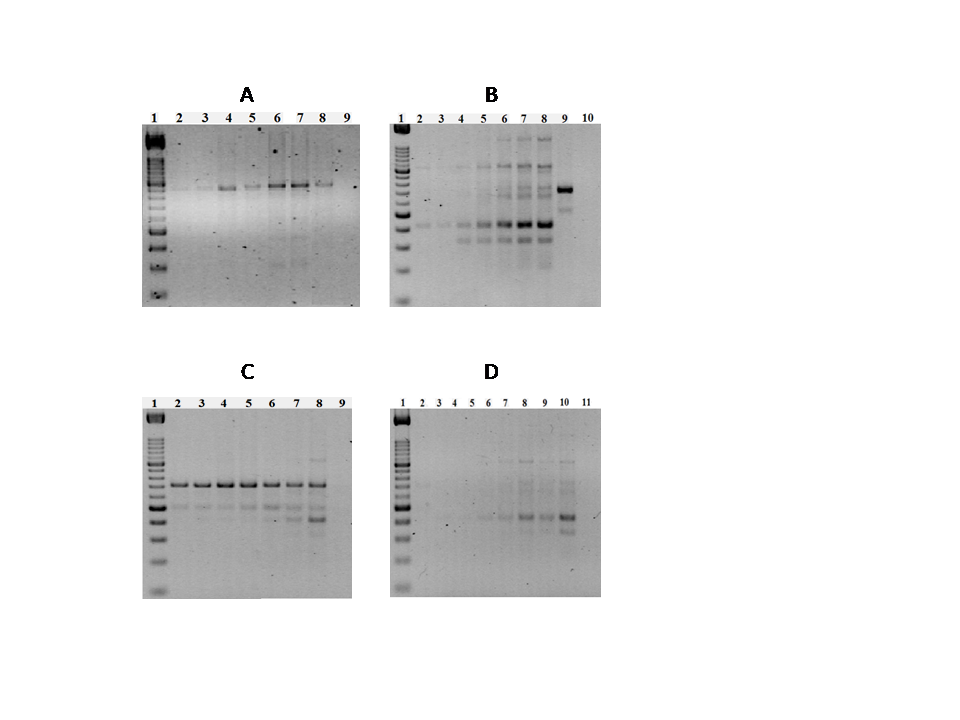

Supplement: Figure S3 — Temperature Gradient for PCR. A: pfhrp2 positive samples. Lane 1: 100 pb plus Molecular marker (MM) - RocheTM; Lane 2: 42.0°C; Lane 3: 43.0°C; Lane 4: 45.0°C; Lane 5: 47.0°C; Lane 6: 48.0°C; Lane 7: 50.0°C; Lane 8: NF54; Lane 9: Negative control (NC) to 50.0°C. B: pfhrp2 negative samples. Lane 1: MM; Lane 2: 50.0°C; Lane 3: 48°C; Lane 4: 47.0°C; Lane 5: 45.0°C; Lane 6: 44.0°C; Lane 7: 43.0°C; Lane 8: 42.0°C; Lane 9: NF54 (50.0°C); Lane 10: NC. C: pfhrp3 positive samples. Lane 1: MM; Lane 2: 50.0°C; Lane 3: 48.0°C; Lane 4: 47.0°C; Lane 5: 45.0°C; Lane 6: 44.0°C; Lane 7: 43.0°C; Lane 8: 42.0°C; Lane 9: NC. D: pfhrp3 negative samples. Lane 1: MM; Lane 2: 50.0°C; Lane 3: 48.0°C; Lane 4: 47.0°C; Lane 5: 45.0°C; Lane 6: 44.0°C; Lane 7: 43.0°C; Lane 8: 42.0°C; Lane 9: 41.0°C; Lane 10: 40.0°C; Lane 11: NC. (0.29 MB TIF) [file pone.0008091.s005.tif]
